# Muscle ultrasound in myopathies

**DOI:** 10.1097/WCO.0000000000001306

**Published:** 2024-07-25

**Authors:** Alex Vicino, Dimitra Veltsista, Nens van Alfen

**Affiliations:** a Nerve-Muscle Unit, Neurology Service, Department of Clinical Neurosciences, Lausanne University Hospital and University of Lausanne, Lausanne, Switzerland; b Department of Neurology, University Hospital of Patras, Patras, Greece; c Department of Neurology& Clinical Neurophysiology, Clinical Neuromuscular Imaging Group, Donders Center for Neuroscience, Radboudumc, Nijmegen, The Netherlands

**Keywords:** biomarker, dystrophy, muscle ultrasound, myopathy, neuromuscular disorder

## Abstract

**Purpose of review:**

This review highlights recent developments in the field of muscle ultrasound (MUS) for the diagnosis and follow up of muscle disorders.

**Recent findings:**

The diagnostic screening capacity of quantitative grayscale analysis is still sufficient to assess children suspected of a neuromuscular disorder. A combination of visual and quantitative assessment is advised for optimal interpretation. MUS was more sensitive but less specific than MRI for detecting pathology in limb girdle dystrophies and inflammatory myopathies. New techniques such as shearwave elastography and artificial intelligence algorithms for automated image segmentation show promise but need further development for use in everyday practice.

Muscle ultrasound has high correlations with clinical measures of function in skeletal and respiratory muscles and the orofacial region, in most of the myopathies and dystrophies studied. Over time, imaging changes precede changes in clinical status, making them attractive for biomarker use in trials. In Duchenne muscular dystrophy MUS was also responsive to the effects of steroid treatment.

**Summary:**

Muscle ultrasound is a sensitive technique to diagnose and follow up of skeletal, facial and respiratory muscles in neuromuscular disorders. Its role is both complementary to and partially overlapping with that of MRI.

## INTRODUCTION

**FIGURE 1:**
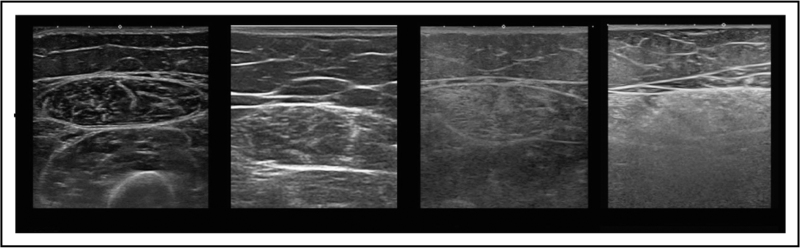
Visual (qualitative) evaluation of muscle ultrasound. Muscle ultrasound of quadriceps (rectus femoris and vastus intermedius) muscle, showing different degrees of muscle abnormalities from left (normal MUS with *starry night* appearance) to right (severe abnormalities). The four images correspond to grades 1 to 4 on the Heckmatt scale, respectively.

**FIGURE 2:**
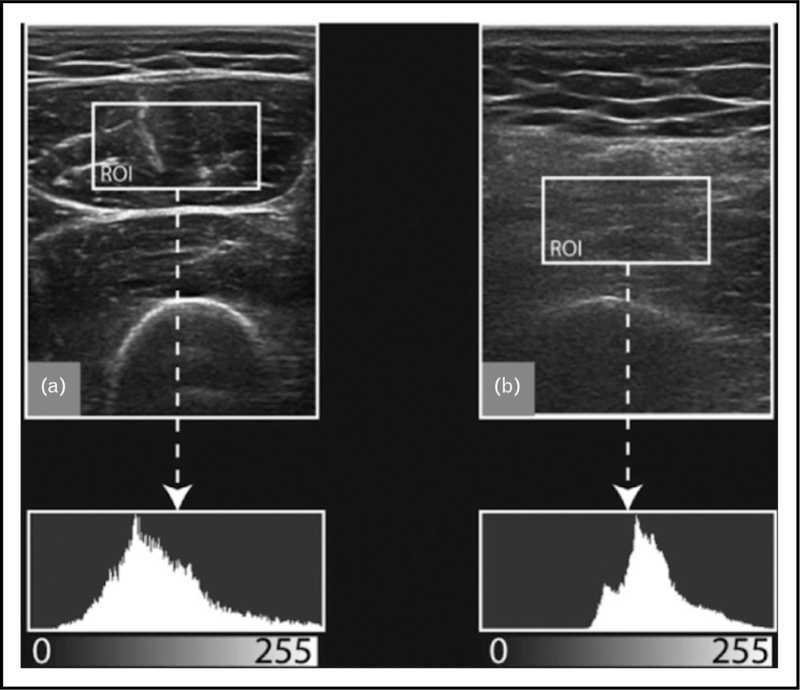
Quantitative MUS analysis. Artificial intelligence analysis tools (e.g. PyRadiomics) allows to extract additional features like texture and shape from the original image: Different features can be combined for further analysis and compared to a database to allow machine learning.

**FIGURE 3:**
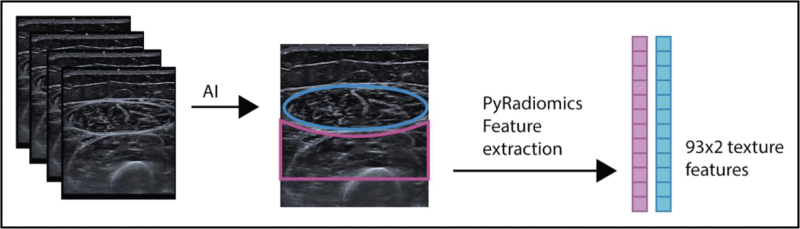
MUS analysis with artificial intelligence. Grayscale histogram analysis of a chosen ROI (=region of interest) in a normal muscle on the left and dystrophic muscle on the right, showing the mean echogenicity value that allows for quantitative MUS analysis.

**FIGURE 4:**
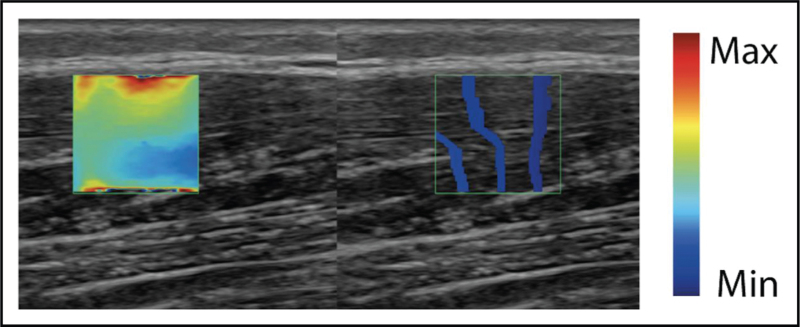
Shear wave elastography. Example of shear wave elastography ultrasound images. A high intensity pulse produces shear waves which propagate laterally. Tracking of laterally propaged shear waves with low intensity pulses allows to measure shear wave velocity, which is a measure of tissue elasticity.

The use of muscle ultrasound for the diagnosis and follow up of myopathies started in the 1980s with two seminal reports on the use of B-mode scanning to diagnose children in realtime [1,2]. Muscle ultrasound, like MRI, is not one technique but a suite of applications ranging from simple B-mode (i.e. brightness mode) imaging to quantified grayscale analysis, tissue texture assessment, elastography, motion capture with speckle tracking, and artificial intelligence (AI)-augmented analysis techniques [3,4,5^▪^] (see Figs. 1–4). 

**Box 1:**
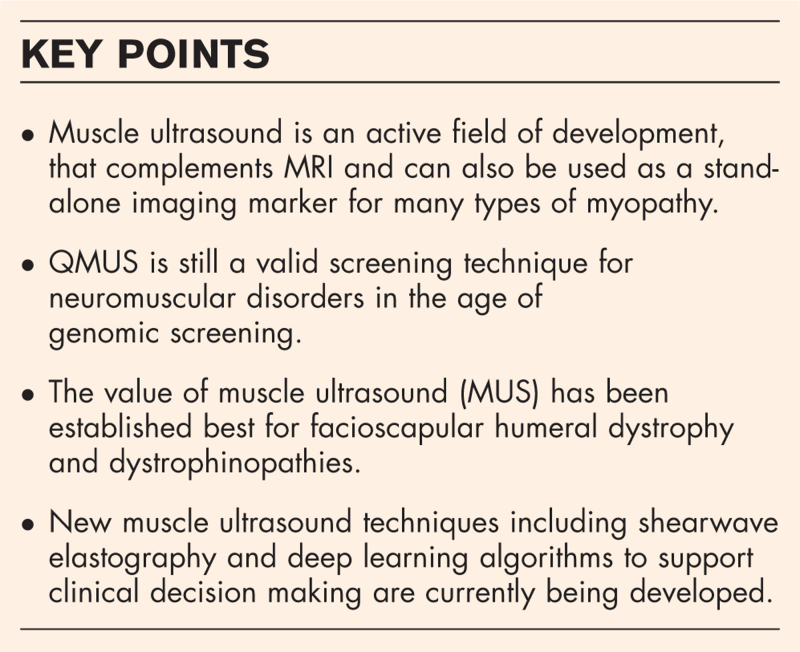
no caption available

B-mode ultrasound with visual and/or quantitative (QMUS) evaluation is the most validated ultrasound application for detecting muscle disorders [6]. Visual analysis by an experienced operator has a 75–85% sensitivity, with higher accuracy in children and in classic myopathies (Fig. 1).

Quantitative grayscale analysis (Fig. 2) using the mean echogenicity value or backscatter analysis is more sensitive (90–95%), but harder to implement because of the need for age, sex and BMI-specific reference values or proprietary manufacturer information [4].

New analysis tools using artificial intelligence (Fig. 3) are being explored to automate and increase detection efficiency in muscle ultrasound [5^▪^].

Dynamic imaging can sensitively assess muscle movement, including fasciculation for which it is currently the most sensitive tool [7]. Muscle shearwave elastography (SWE) (Fig. 4) is a more recent ultrasound application, that explores changes in tissue stiffness in several dystrophies and myotonias.

Muscle imaging techniques are used for diagnostic screening, pattern recognition and follow up. Imaging can guide more invasive tests such as needle electromyography and muscle biopsy and provide context information for genetic variants of unknown significance [8].This review describes recent developments in the field of muscle ultrasound, and provides suggestions for the use of the technique in the neuromuscular clinic and research.

## GENERAL ROLE OF MUSCLE ULTRASOUND, AND TECHNIQUE DEVELOPMENT

### Diagnostic screening value

Initial diagnostic screening studies of muscle ultrasound (MUS; Figs. 1 and 2) showed a high sensitivity and specificity of ≥90% [9], a finding corroborated by a recent meta review [10]. A prospective study reviewed the value of QMUS screening in the age of genomic sequencing, assuming that classic genetic NMDs would nowadays be more easily identified [11], skewing the referrals for screening towards patients with less obvious pathologies. The QMUS sensitivity (83%) and specificity (78%) were slightly lower than two decades ago, but still sufficient to use QMUS for screening.

### Dysphagia assessment

Suprahyoid muscle ultrasound with B-mode and M-mode in neuromuscular and stroke patients [12^▪^] found significantly different muscle displacement during a standardized swallow between healthy persons and patients with mild or severe dysphagia, with a calculated sensitivity of 93.3%, a77.8% positive predictive value and a 95.6% negative predictive value. Ultrasound constituted an improvement over the routine clinical assessment.

### Orofacial muscle ultrasound

A strong correlation of ultrasound with chewing and swallowing quality was found using orofacial muscle ultrasound in healthy people and patients suspected of a neuromuscular disorder (NMD) [13]. Rater experience increased the reliability of visual analysis, and condensing the 4-point Heckmatt grading scale to a 3 points scale resulted in easier applicability of visual analysis in practice.

### Effects of physical fitness on adipose tissue and muscle ultrasound

A significant inverse correlation between physical fitness and muscle echogenicity and adipose tissue was found in almost 300 children aged 5–9 years [14], underpinning the association of more contractile muscle tissue with higher physical activity levels. In children with progressive NMD, the physical fitness level could affect the offset and slope of echogenicity changes, which deserves consideration when using imaging to inform muscle status in a trial.

### Muscle ultrasound image formation

Attenuation of the ultrasound beam [15] decreases muscle echogenicity in the deeper muscle layers in muscle dystrophy patients [16]. Practically this means that the region of interest for MUS analysis should be limited to the superficial muscle parts in patients with advanced disease [17]. A comparison of almost 14 000 muscle ultrasound images evaluated visually and quantitatively [6] showed that these methods are complementary rather than interchangeable, with a positive correlation of 0.7 in myopathies. Advanced fatty degeneration led to clearly abnormal muscles visually and a decrease rather than further increase in echogenicity, i.e. a parabolic rather than a linear correlation. The authors suggested to always combine visual with quantitative assessment.

### Shearwave elastography

SWE is an adjunct to structural muscle imaging, mainly explored in Duchenne muscular dystrophy [3,18,19,20] (Fig. 4), where it was less sensitive to change than echogenicity and sound attenuation measures [21]. SWE values depend critically on the muscle studied and the ultrasound equipment used [22] and need machine specific references for their interpretation. In addition, standardized scan protocols are needed for reliable assessment [23], that also incorporate patient status (at rest, after activity, etc.). Only superficial muscle regions can be assessed. More research focusing on the reliability of SWE will be needed before it can be applied in the clinic.

### Artificial intelligence in muscle ultrasound

A narrative review evaluated 12 studies that explored the diagnostic use of AI algorithms [24] (Fig. 3) in neuromuscular disease using ultrasound, MRI, EDx and genetics, and found that their classification was already ≥90% accurate [5^▪^]. This was corroborated by a study showing 94% accuracy for classifying DMD patients by their ambulatory capacity [25]. AI algorithms need careful construction and assessment, as there is a risk of overfitting their predictive capabilities when trained on a selected set of images and patients, which impairs their use in unselected cohorts. This means that practical AI implementation from research to the clinic will require additional effort across the field to make its use safe and accountable [26].

### Muscle ultrasound comparison with other diagnostic techniques

A study comparing MUS with time-matched needle EMG of 800 muscles from NMD patients [27^▪▪^] found that the techniques showed disparate results more often than not, indicating a complementary rather than a comparable role. Either technique could be used reliably to exclude pathology. Muscle ultrasound performed best in the detection of typical myopathies and was less sensitive for mild neurogenic abnormalities. Two studies comparing muscle ultrasound and MRI in FSHD [28,29] corroborated the tight cross-sectional correlation between clinical data, muscle echogenicity and MRI fatty replacement. A 5-year longitudinal follow up found that normal muscles at baseline more often showed an echogenicity increase (in 17%) than an MR fat fraction increase (in 10%), confirming that ultrasound seems better suited for early disease, while MRI is better at capturing late-stage changes [30].

## HEREDITARY MYOPATHIES

Despite the exponential advances in MUS with several hundred peer-reviewed studies published to date, research in hereditary myopathies has mostly focused on dystrophinopathies, followed by FSHD. Literature regarding other rare myopathies is limited to few case reports and small series. Nonetheless, several characteristic US findings and patterns have been reported, such as the selective sparing of triceps muscles in Pompe Disease and ‘central clouding’ of rectus femoris in COL6 related congenital muscular dystrophy [31], but an extensive survey of this topic is beyond the scope of this article that presents recent findings.

### Dystrophinopathies

In Duchenne muscular dystrophy (DMD )MUS shows a homogeneous muscle echogenicity increase, typically prior to clinical weakness and before a motor development plateau occurs [31]. Initially, adductor magnus and gastrocnemius are involved [32], with most progression in the rectus femoris in younger boys [33]. QMUS changes in rectus femoris correlated significantly with clinical assessments in ambulant, steroid-naïve patients [34], and in corticosteroid-treated patients where QMUS was found to [35] to be responsive to treatment [36]. Orofacial muscles become abnormal early in dystrophinopathies, with no relation the ambulatory status, and predict mastication and swallowing problems [37^▪^]. Tongue and temporalis muscle ultrasound shows pseudohypertrophy in DMD and BMD [37^▪^,^38^]. A strong correlation between spirometry and diaphragm thickening but a weak correlation with diaphragm thickness was found in 74 DMD patients [39].

### Fascioscapulohumeral dystrophy

The typical asymmetric muscle involvement in fascioscapulohumeral dystrophy (FSHD) was confirmed with MUS [40]. Trapezius, rectus femoris and rectus abdominis were most commonly affected, with a heterogenous and multifocal hyperechogenicity pattern [31]. MUS showed abnormalities in 94% of symptomatic patients, with strong correlations with symptom status and echogenicity change over time, confirming its biomarker potential [41^▪^]. Changes in muscle echointensity changes were more important than changes in clinical assessments over 12 months [42].

### Limb-girdle muscular dystrophy

The consistency between MUS and muscle MRI for detecting limb-girdle muscular dystrophy (LGMD) subtypes was 80%, with superior sensitivity and accuracy of MUS and higher specificity using MRI, suggesting they might be used interchangeably [40].

### Oculopharyngeal muscular dystrophy

MUS performed bilaterally in 11 orofacial and limb muscles, detected significant echogenicity changes in the temporalis, tongue, and deltoid, and diameter decrease in 7/11 muscles measured (temporalis, masseter, digastric, tongue, deltoid, iliopsoas, and soleus), over a period of 20 months [43].

### Congenital muscular dystrophy

An observational QMUS study in11 SELENON (SEPN1)-related congenital myopathy patients showed symmetric involvement, most prominently of the sternocleidomastoid and biceps brachialis, while soleus and temporalis were least affected. Diaphragm US displayed severe atrophy and decreased contraction [44]. MUS in LAMA2-related muscular dystrophy showed symmetric involvement, most severely of the sternocleidomastoid and biceps brachialis, while the soleus was least affected; contrasting earlier MRI work. The authors proposed scanning rectus abdominis, vastus lateralis and gastrocnemius as the most responsive outcome measures [45]. MUS performed inFHL1-related reducing-body myopathy showed heterogeneous and focally increased echogenicity in 11/18 patients, with selective involvement of deeper layers of the biceps and tibialis anterior, corroborated by muscle MRI [46].

### Congenital myopathies

A MUS study in 40 RYR1-related malignant hyperthermia patients showed frequent abnormalities, most often muscle hypertrophy and increased echogenicity, corroborating previous reports [47].

### Metabolic/mitochondrial myopathies

Mitochondrial myopathy patients usually become symptomatic because of an energy deficiency rather than structural muscle pathology. MUS imaging can be normal in the majority of children in the early stages [48]. In contrast, glycogen storage disorders show prominent MUS abnormalities [49]. MUS was proven useful for newborn screening of infantile onset Pompe disease, and to measure outcome in late onset Pompe disease (LOPD) [50]. Muscle echointensity changes were most pronounced in the thoracic paraspinals, quadriceps, medial gastrocnemius, tibialis anterior and biceps brachii [51]. Echo heterogeneity index, but not the shear modulus, correlated strongly negatively with qualitative motor function measures of the lower limbs, with relative sparing of triceps brachii [52^▪^]. There was a good correlation between echointensity increase and clinical motor function, and increased echogenicity of the abdominal and trunk muscles combined with sparing of the biceps and flexor digitorum muscles could distinguish LOPD from DM1 [53]. Involvement of the tongue was more pronounced in LOPD compared to other acquired and hereditary myopathies [54].

## TOXIC AND IATROGENIC MYOPATHIES

### Glucocorticoid-induced myopathy

A literature review concluded that evidence supports the use of MUS as a diagnostic and monitoring tool for glucocorticoid (GC)-induced myopathy [55]. Preliminary studies suggest that increased muscle echointensity is more pronounced in the tibialis anterior, and correlates with treatment duration, while atrophy is more prominent in proximal muscles [56,57^▪^].

### Intensive care unit acquired weakness

Several studies have explored the possible diagnostic use of MUS in intensive care unit-acquired weakness (ICU-AW; critical illness neuromyopathy). Common MUS findings are a decrease in muscle CSA and thickness, increase in muscle echointensity and decrease in pennation angle, most obvious in anterior thigh muscles [58]. Muscle echogenicity changes were found from the first day of ICU admission [59]. Novel methods including SWE, superb microvascular imaging and contrast-enhanced ultrasound in ICU-AW patients versus healthy controls also showed significant qualitative changes [60]. Visual evaluation was found superior to quantitative analysis in predicting ICU-AW, with a sensitivity of 86% and a specificity of 60%, and also for discriminating patients with ICU-AW from those without [61^▪^]. A higher reduction rate of the rectus femoris pennation angle was the best predictor of ICU-AW, compared to rectus femoris CSA, diaphragm and intercostals muscles thickness measurements [58]. A study of changes in muscle mass during the first week of ICU stay, demonstrated significantly higher mortality rates in patients with muscle enlargement, than those with muscle wasting [62].

## MYTONIC MYOPATHIES, CHANNELOPATHIES, IDIOPATHIC INFLAMMATORY MYOPATHIES AND IMMUNE CHECKPOINT MYOSITIS

### Idiopathic inflammatory myopathies

Evidence is accumulating for a role of MUS in the diagnosis and follow up of idiopathic inflammatory myopathies (IIM) [63]. However, the literature is hampered by the use of the outdated and imprecise categorization of “polymyositis”, that has been replaced by more precise entities: immune-mediated necrotizing myopathies (IMNM), connective tissue disease overlap syndrome (OS), antisynthetetase syndrome (ASS), and inclusion-body myositis (IBM). Dermatomyositis (DM), and its juvenile form (JDM) have been maintained as separate disorders [64,65]. Several studies have explored MUS to diagnose IIM overall. Increased echogenicity showed a good correlation with muscle atrophy on MRI, and an overall good sensitivity but very low specificity for predicting MRI and histopathologic abnormalities [66]. Muscle SWE velocity was significantly lower in the quadriceps and hamstring muscles in IIM compared to healthy controls [67], a finding confirmed in subsequent studies that also demonstrated higher echogenicities in IIM versus healthy persons [68]. Lower SWE velocity was associated with edema and atrophy on MRI (with variation depending on the examined muscle), without a correlation with disease duration or CK levels [67].

MUS has been used to study the effect of immunomodulatory treatment. Qualitative and Heckmatt-scale abnormalities were decreased after 9 weeks of intravenous immunoglobulin treatment, while quantitative MUS and MRI parameters did not show any changes [69]. In JDM, echogenicity and muscle thickness of the rectus femoris could discriminate between patients and healthy controls, and had a good correlation with functional parameters, MRI abnormalities, and changes in power Doppler before and after exercise [70].

### Inclusion body myositis

Quantitative echogenicity and muscle thickness are able to discriminate IBM from other IIM and other neuromuscular diseases [71], a finding confirmed in a recent meta-analysis that showed a pooled sensitivity of 82% and specificity of 98% to discriminate IBM from controls. No difference was found for muscle thickness [72]. In the latest IBM diagnostic criteria, increased echogenicity of the flexor digitorum communis, vastus medialis and lateralis and medial gastrocnemius have been included, along with muscle MRI and anticN1A antibodies, as tools to support the diagnosis [73^▪▪^].

Respiratory MUS found the diaphragm thickening fraction correlated with muscle strength, disease duration, and the dyspnea score, while muscle strength did not [74]; which could help detect respiratory impairment earlier.

### Immune-mediated necrotizing myopathy

MUS in 6 patients with anti-HMGC-related IMNM showed a heterogenous and patchy muscle echogenicity increase that became more diffuse with advanced disease. In the upper limbs MUS revealed a selective involvement of biceps brachii more than the triceps [75].

### Immune-checkpoint related inflammatory myopathy

Myositis is the most frequent neurologic complication of immune checkpoint inhibitor (ICI) immunotherapy [76]. Imaging with PET-CT and MRI is useful to detect subclinical involvement and inform the differential diagnosis [77]. Respiratory involvement is a potentially lethal complication of ICI myositis; reduced diaphragm thickness at the end of expiration and absence of thickening with inspiration were reported as MUS markers of respiratory impairment [78].

### Skeletal muscle channelopathies

Myotonia is one of the core symptoms of channelopathies, together with transient impairment of voluntary contraction (i.e. periodic paralysis) [79]. Myotonia quantification would be a welcome outcome for treatment trials. Its duration was quantified with B-mode MUS [80] or with SWE for during and after contraction [81], although these finding need reproducing in other patients.

### Myotonic dystrophies

Myotonic dystrophy type 1 (DM1) could be distinguished from IBM and other IIM by texture analysis with 76% accuracy. This image postprocessing approach, that characterizes the spatial relationship and echogenicity variation of adjacent pixels, showed less pixel variability in the DM1 patients [82]. Respiratory impairment was studied with diaphragm ultrasound in DM1, and found that diaphragm thickness was lower in DM1 patients than healthy persons, accompanied by a reduced thickening ratio. This thickening ratio was also correlated with more severe clinical impairment on the muscular impairment rating scale [83].

## CONCLUSION

Muscle ultrasound for neuromuscular disorders is an active field of research and development. Its role is partially overlapping and also complementary to that of MRI. The current evidence suggests that MUS can play a role as a diagnostic and follow up marker for skeletal, facial and respiratory muscles across the various types of myopathies. New techniques including SWE and AI algorithms to support decision making are currently under development.

## Acknowledgements

*None.*

### Financial support and sponsorship

*None.*

### Conflicts of interest

*Nens van Alfen is an ultrasound instructor for Sonoskills and performs editorial services for Wiley Publishing; all payment goes to their employer. Alex Vicino and Dimitra Veltsista have none.*

## REFERENCES AND RECOMMENDED READING

Papers of particular interest, published within the annual period of review, have been highlighted as:

▪ of special interest
▪▪ of outstanding interest
